# Molecular dissection of *Phaseolus vulgaris* polygalacturonase-inhibiting protein 2 reveals the presence of hold/release domains affecting protein trafficking toward the cell wall

**DOI:** 10.3389/fpls.2015.00660

**Published:** 2015-08-26

**Authors:** Monica De Caroli, Marcello S. Lenucci, Francesca Manualdi, Giuseppe Dalessandro, Giulia De Lorenzo, Gabriella Piro

**Affiliations:** ^1^Dipartimento di Scienze e Tecnologie Biologiche ed Ambientali, Università del SalentoLecce, Italy; ^2^Dipartimento di Biologia e Biotecnologie Charles Darwin, Università degli Studi di Roma “La Sapienza”Rome, Italy

**Keywords:** cell-wall trafficking, PGIP2, secretion pathway, cell wall protein, GFP, Sp2 independent secretion

## Abstract

The plant endomembrane system is massively involved in the synthesis, transport and secretion of cell wall polysaccharides and proteins; however, the molecular mechanisms underlying trafficking toward the apoplast are largely unknown. Besides constitutive, the existence of a regulated secretory pathway has been proposed. A polygalacturonase inhibitor protein (PGIP2), known to move as soluble cargo and reach the cell wall through a mechanism distinguishable from default, was dissected in its main functional domains (A, B, C, D), and C sub-fragments (C1–10), to identify signals essential for its regulated targeting. The secretion patterns of the fluorescent chimeras obtained by fusing different PGIP2 domains to the green fluorescent protein (GFP) were analyzed. PGIP2 *N*-terminal and leucine-rich repeat domains (B and C, respectively) seem to operate as holding/releasing signals, respectively, during PGIP2 transit through the Golgi. The B domain slows down PGIP2 secretion by transiently interacting with Golgi membranes. Its depletion leads, in fact, to the secretion via default (Sp2-susceptible) of the ACD-GFP chimera faster than PGIP2. Depending on its length (at least the first 5 leucine-rich repeats are required), the C domain modulates B interaction with Golgi membranes allowing the release of chimeras and their extracellular secretion through a Sp2 independent pathway. The addition of the vacuolar sorting determinant Chi to PGIP2 diverts the path of the protein from cell wall to vacuole, suggesting that C domain is a releasing rather than a cell wall sorting signal.

## Introduction

Plant cell wall is a highly dynamic extracellular compartment whose synthesis and remodeling occurs continuously during cell growth and differentiation and in response to biotic and abiotic stresses. This implies a continuous delivery of *de novo* synthesized molecules to the cell wall through the cell secretory system. The different nature of the main cell wall components implies their compartmentalized synthesis that involves plasma membrane for cellulose, Golgi apparatus for matrix polysaccharides (pectins and hemicelluloses), endoplasmic reticulum for structural and enzymatic proteins, comprising the hexameric cellulose synthase complexes. Cell wall proteins and matrix polysaccharides are modified during their journey along the secretory pathway, packaged into vesicles and exported to the plasma membrane where they are released and integrated into the microfibrillar structure of cell wall (Somerville, 2006; Driouich et al., 2012; McFarlane et al., 2014).

The molecular mechanisms that control the trafficking of cell wall components are still poorly understood, but a growing body of evidence suggests a model more complex than previously thought. With respect to this, two new interesting aspects are emerging in recent years: (i) the discovery of unconventional protein secretion (UPS) (Ding et al., 2012, 2014) and (ii) the accumulation of evidence contrasting the notion that secretion to cell wall occurs by default (Wolf et al., 2009a,b; De Caroli et al., 2011a).

UPS consists in the secretion of proteins lacking an usual *N*-terminal leader peptide responsible for ER targeting. Combined biochemical, proteomic and bioinformatic analyses revealed that these leaderless secretory proteins (LSPs) contribute for more than 50% to the total extracellular proteins, indicating UPS as complementary to the conventional ER/Golgi secretion pathway (Agrawal et al., 2010). A close connection has also been proposed between UPS of LPSs and the recently discovered plant EXPO (exocyst-positive organelle) compartment (Wang et al., 2010).

Secretion to the apoplast has always been considered a default pathway (Jurgens, 2004). This was supported by the evidence that the fusion with the signal peptide for ER translocation is sufficient for the secretion of non-plant soluble proteins such as secGFP and secRGUS (a modified variant of rat preputial β-glucuronidase that is efficiently secreted) (Denecke et al., 1990; Batoko et al., 2000). The bulk flow secretory pathway of GFP variants involves the SNARE SYP121, and is inhibited by Sp2, a dominant-negative truncated soluble form of SYP121 (Geelen et al., 2002; Leucci et al., 2007; Rehman et al., 2008). Although no specific determinants have been yet identified in protein secreted to the plasma membrane and cell wall, active sorting processes of the vesicles and their cargo have been reported. During cytokinesis, the cell plate is built up through a very polarized trafficking of Golgi-derived vesicles that appears as a form of regulated secretion (Bassham et al., 2008). Further, examples of polarized secretion are also well known for Golgi-derived vesicles targeted to specific plasma membrane regions at the site of microbial attack. Local release of pathogenesis-related proteins as well as structural and chemical remodeling of the cell wall at sites of infection are crucial for resistance to pathogen penetration (Hückelhoven, 2007). Significantly, specific syntaxins seem to be involved in these events such as KNOLLE (SYP111) and PEN1 selectively expressed during the cell plate formation (Jürgens, 2005) and the fungal attack (Assaad et al., 2004), respectively.

Studies on the secretion of several cell wall proteins and polysaccharides have revealed the existence of undefined Golgi stop signals, of cell wall secretion pathways distinguishable from the default sorting, and of *post*-Golgi compartments involved in the transport to the apoplast, pointing to an unexpected complexity of the process. Indeed, it has been reported that: (i) before reaching the apoplast, several cell wall proteins are retained in the Golgi stacks until specific signals at the *N*-terminus are proteolitically removed (Dal Degan et al., 2001; Wolf et al., 2009a,b); (ii) the SNARE SYP121, involved in the bulk flow secretory pathway, does not participate to the secretion into the apoplast of matrix polysaccharides (Leucci et al., 2007; Rehman et al., 2008; Silva et al., 2010), *Phaseolus vulgaris* polygalacturonase-inhibiting protein 2 (PGIP2) and *Arabidopsis* pectin methylesterase inhibitor protein 1 (PMEI1) (De Caroli et al., 2011a); (iii) in *Arabidopsis*, rice and tobacco BY-2 cells, a secretory vesicle cluster (SVC) containing soluble proteins and cell wall components appears as a novel motile compartment, distinguished from Golgi stacks and TGN, involved in mass secretion of cell wall material (Toyooka et al., 2009).

In particular, the GFP tagged form of PGIP2, a protein involved in plants protection against pathogens (Kalunke et al., 2015), is a useful tool for monitoring cell-wall protein transport through the endomembrane system. PGIP2-GFP moves as a soluble cargo using secretory mechanisms not affected by Sp2, the dominant-negative truncated soluble form of SYP121 (De Caroli et al., 2011a). In this work, we fragmented PGIP2 in its main domains and analyzed their secretion patterns fusing them to GFP. Our results suggest a specific role for the *N*-terminus and leucine-rich repeat (LRR) domains, which seem to act respectively as holding/releasing signals during the transit of PGIP2 through Golgi stacks.

## Materials and methods

### Plasmid construction

Oligonucleotides used for cloning are listed in Table S1. PGIP2-GFP plasmid was obtained as described in De Caroli et al. (2011a). The PGIP2-GFP plasmid was amplified with primers P1 and P2 using the QuikChange® Site-Directed Mutagenesis Kit (Stratagene, Agilent Technologies, http://www.chem.agilent.com/) to introduce a *Nhe*I restriction site between the region encoding A and B domains. The *Nhe*I digested PGIP2-GFP plasmid, without the region encoding B, C and D domains, was re-ligated on itself, obtaining A-GFP plasmid. In the construct we included the leader sequence from nucleotide + 1 to +27 of *pgip1* that was shown to drive high level expression of *pgip2* (Devoto et al., 1998). To obtain AB-GFP plasmid, a *Nhe*I restriction site was introduced between the region encoding B and C domains of PGIP2-GFP plasmid using primers P3 and P4. ABC-GFP plasmid was obtained by inserting the region encoding A, B, and C domains into PGIP2-GFP (primers P5 and P6). Similarly, using primers listed in Table S1 the constructs containing increasing length of C sub-fragments (from ABC_1_-GFP to ABC_10_-GFP) were obtained. A plasmid containing ACD domains was synthesized by Eurofins Genomics s.r.l (http://www.eurofinsgenomics.eu). It was inserted into PGIP2-GFP plasmid as a *BamH*I/*Nhe*I fragment to obtain ACD-GFP. A-GFP was cut with *Xho*I/*Nhe*I and cloned in Aleu-RFP plasmid (De Caroli et al., 2011b) to obtain A-RFP plasmid. PGIP2-RFP plasmid was obtained by inserting PGIP2 into Aleu-RFP plasmid as a *BamH*I/*Nhe*I fragment. All constructs were checked by sequencing (Eurofins Genomics s.r.l.).

### Protoplast preparation and transformation

Tobacco leaf protoplasts were prepared and transformed as described by Leucci et al. (2007). Unless otherwise stated, equal quantities (20 μg) of each plasmid were used for transformation, co-localization and co-expression experiments.

### *In vivo* immunolabeling of proteins on protoplast surface

Protoplasts expressing ABC-GFP, ABC_*1–4*_-GFP, and ABC_*1–5*_-GFP were immunolabeled with anti-GFP serum (A6455, Molecular Probes, http://www.invitrogen.com/) and Alexa Fluor 546-conjugated anti-rabbit IgGs (Molecular Probes) as reported in De Caroli et al. (2011a).

### Drug treatments

Treatment with cycloheximide (Sigma-Aldrich, http://www.sigmaaldrich.com) was performed by incubating transformed protoplasts (22 h after transformation) with cycloheximide (30 μg·mL^−1^) in K3 medium (Freydl et al., 1995) for 2 h before confocal analysis. Tunicamycin (100 mg·mL^−1^; Sigma-Aldrich) was added to the K3 medium after protoplast transformation and was present for the entire incubation time (24 h).

### Confocal laser scanning microscopy

Protoplasts transiently expressing fluorescent constructs were observed by a confocal laser scanning microscope (LSM 710, Zeiss, http://www.zeiss.com/) in their culture medium at different times after transformation. Observations were performed as described in De Caroli et al. (2014).

### Protein extraction

Protoplasts were harvested by gentle centrifugation at 65 g without break, after dilution of the incubation medium with two volumes of W5 (Freydl et al., 1995). The medium was concentrated by filtration on Cetricon Plus 20 (Amicon, http://www.millipore.com/) to obtain the extracellular protein fraction. Protoplasts were suspended in homogenization buffer and sonicated as reported in Piro et al. (2000). The homogenate was centrifuged at 800 g for 10 min at 4°C and the supernatant was precipitated with 80% acetone at −20°C (three times) to obtain protein intracellular fraction. The 800 g pellet, consisting of cell walls and cell debris, was sequentially treated with 1 ml of homogenization buffer (three times), 1 ml of chloroform/ methanol (1/1 v/v; 10 times) and acetone (three times) (Lenucci et al., 2006). Non-covalently bound cell-wall proteins were extracted from delipidated cell walls as reported in Leone et al. (2000). The amount of proteins in each fraction was spectrophotometrically determined according to Bradford quantification method (Bradford, 1976). An identical amount of proteins (10 μg/line) of the three subcellular fractions was subjected to SDS-PAGE and Western Blotting. Each experiment was independently repeated three times for each chimera.

### Cell lysis and phase separation

Transformed protoplast pellets (65 g) were freeze/thawed twice in 10 mM Tris-buffer pH 7.5 containing 0.15 M NaCl and 1 mM EDTA and centrifuged at 800 g for 10 min to remove cell walls; the supernatant was then adjusted to 1.5 ml using Tris-buffer, and Triton X-114 was added to a final concentration of 1% v/v. Phase separation was carried out as described by De Caroli et al. (2014).

### SDS-PAGE and Western blotting

SDS-PAGE and Western blotting were carried out as described in De Caroli et al. (2011a). Anti-GFP (1:5000 v/v) (Molecular Probes) and anti-Sp1 rabbit polyclonal serum (1:5000 v/v) (Geelen et al., 2002) were used in TBS + 1% skimmed-milk powder. Protein bands were detected with ECL™ Western Blotting Analysis System (GE Healthcare, http://www.gehealthcare.com) and their intensity was measured by using an image analyzer (Kodak EDAS 290) and the software 1D 3.6.

### Pulse-chase and immunoprecipitation

For pulse-chase labeling of transformed protoplasts 15 MBq ml^−1^ of EasyTag (TM) EXPRE35S35S Protein Labeling Mix, [^35^S] (Perkin Elmer Italia S.P.A.) were using following Leucci et al. (2007). Immunoselected proteins were analyzed by SDS-PAGE and fluorografy.

### Standard RNA procedures and quantitative real-time PCR

Total RNA was extracted from transformed protoplasts using SV Total RNA Isolation System according to the manufacturer's instructions (PROMEGA). The RNA of each sample was reverse transcribed using oligo (dT) primer with TaqMan® Reverse Transcription Reagents (Applied Biosystems, http://www.lifetechnologies.com/) according to the manufacturer's standard protocol. The obtained cDNAs were quantified using a NanoDrop® ND-1000 UV-vis spectrophotometer, diluted, and used for Real-Time PCR amplifications with specific primers (Table S1). Quantitative real-time PCR was performed using SYBR Green fluorescent detection in a Real-Time PCR thermal cycler (ABI PRISM 7700 Sequence Detection System, Applied Biosystems) as accurately described in Aprile et al. (2011). The PCR programme was as follows: 3 min at 94°C; 35 cycles of 30 s at 94°C, 30 s at 60°C, and 30 s at 72°C; and 6 min at 75°C. The specificity of PCR products was checked by performing a melting-curve test.

### Statistical analyses

In three independent experiments, the percentage secretion after Sp2 treatment was normalized with respect to the most efficient secretion value of the control (set to 100%) (Leucci et al., 2007). Data were then statistically analyzed using a One-Way ANOVA test in SigmaStat software (Systat Software Inc., http://www.systat.com/). The Holm-Sidak *post-hoc* method was used to establish significant differences between means with a confidence level of at least 95% (Glantz, 2002).

## Results

PGIP2 exhibits four functional domains named A, the signal peptide; B, the *N*-terminus of the mature protein; C, which comprises 10,5 LRRs; D, the *C*-terminus (Leckie et al., 1999; Di Matteo et al., 2003). To search for a specific sequence responsible for the targeting to the cell wall, a series of fluorescent chimeras were constructed by fusing GFP to the individual A, B, C domains and to C sub-fragments (Figure S1). The fluorescent constructs were transiently expressed in tobacco protoplasts and analyzed by confocal laser scanner microscopy and biochemical techniques.

### The A domain of PGIP2 directs GFP into the secretory system

**Figure 1:**
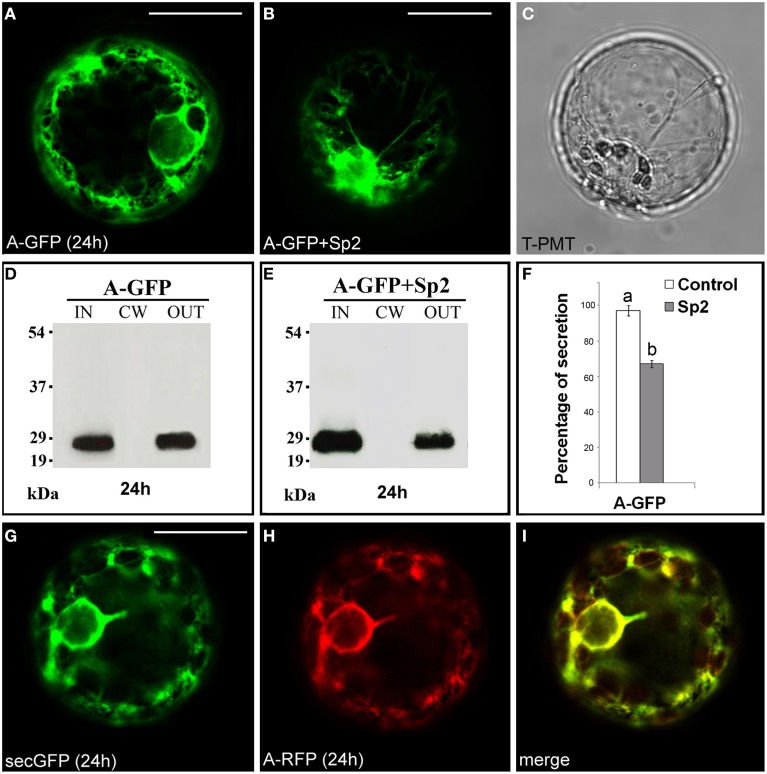
**Transient expression of A-GFP in tobacco protoplasts. (A)** A-GFP labeled the endomembrane system and **(B)** accumulated within protoplast in the presence of Sp2. **(C)** Transmitted light detector (T-PMT) image of **(B)**. **(D)** Western blot of proteins obtained from the intracellular (IN), cell wall (CW) and incubation medium (OUT) fractions of A-GFP-expressing protoplasts. **(E)** Western blot showing the effect of Sp2 on A-GFP secretion. Bands were detected using an anti-GFP serum. **(F)** Quantification of the effect of Sp2 co-expression on the secretion of A-GFP. Data are means ± standard deviation of three independent experiments, normalized with respect to highest value (set to 100%) observed in the control. Different letters indicate significant differences between treatments within each protein group (Holm-Sidak test, *P* < 0.05). **(G)** SecGFP and **(H)** a red variant of A-GFP (A-RFP) showed an overlapping secretion pattern **(I)**. The same fluorescent pattern was observed in 97 ± 2% among over 300 transformed protoplasts examined (100 for each of three independent transformations). Scale bars = 20 μm.

The fusion A-GFP (Figure S1) labeled the endomembrane system (Figure 1A) showing a fluorescence pattern very similar to that of the secreted GFP variant secGFP (Leucci et al., 2007). Western blot of intra- (IN), cell wall (CW), and extra-cellular (OUT) proteins showed the presence of a 27 kDa band, corresponding to GFP molecular mass, in the IN and OUT fractions. No bands were detected in the CW fraction (Figure 1D). When A-GFP and Sp2 were co-expressed in tobacco protoplasts, an accumulation of the chimera within the protoplast was observed (Figure 1B), and a significant (*P* < 0.05) decrease in the amount of the protein in the OUT fraction was detected (Figures 1E,F) indicating that the chimeric protein is secreted through the default pathway. To validate these data, the red variant of the chimera A-RFP and secGFP were co-expressed in tobacco protoplasts. A complete overlapping of the two fluorescence patterns was observed (Figures 1G–I). Together, these results confirm that the A domain represents the signal peptide of PGIP2 that directs the nascent protein into the ER for subsequent secretion, similarly to the secretion signals of secGFP and secRGUS. Likely, no other localization signals are present in this domain since the chimera is secreted into the medium by default.

### The B domain leads to retention of GFP in the Golgi stacks

**Figure 2:**
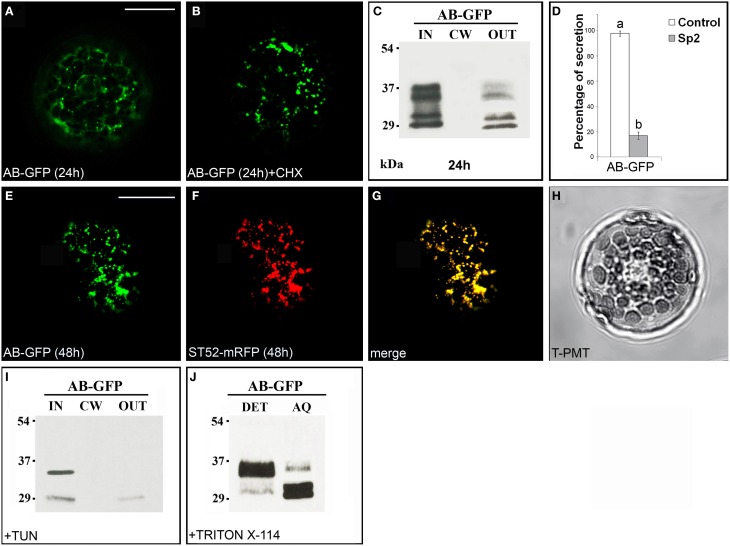
**The B domain blocks AB-GFP in the Golgi stacks. (A)** Approximately 24 h after transformation, AB-GFP transiently expressed in tobacco leaf protoplasts labeled ER and Golgi stacks. **(B)** Cycloheximide (CHX) treatment (30 μg · mL^−1^) of AB-GFP transformed protoplasts evidenced the final localization of the protein in the Golgi stacks. The punctate staining pattern was observed in 98 ± 2% among over 300 protoplasts examined. **(C,I)** Western blot analysis of protein fractions (intracellular, IN; cell wall, CW; incubation medium, OUT) obtained from AB-GFP transformed protoplasts **(C)** and from AB-GFP protoplasts treated with 100 μM tunicamycin for 24 h **(I)**. **(D)** Effect of Sp2 on the secretion of AB-GFP. Statistical analysis is as in Figure 1. **(E)** After 48 h of transformation AB-GFP still labeled Golgi stacks as evidenced by co-localization with the Golgi marker ST52-mRFP **(F)**. **(G)** Merged image of **(E,F)**. The co-localization pattern was observed in 97 ± 2% among over 300 transformed protoplasts examined. Three independent transformation experiments were performed, and approximately 100 transformed protoplasts were counted each time. **(H)** Transmitted light detector (T-PMT) image of **(E–G)**. **(J)** Detergent extraction of AB-GFP transformed protoplasts showing that the full-length form of AB-GFP is Triton X-114 insoluble (detergent phase, DET) while the degraded form is soluble and recovered in the aqueous phase (AQ). Bands were detected using anti-GFP serum. Scale bars = 20 μm.

The chimera AB-GFP (Figure S1), transiently expressed in tobacco leaf protoplasts, was found in the ER and in the Golgi stacks after 24 h (Figure 2A). A 2 h treatment with the protein synthesis inhibitor cycloheximide, which leads to the disappearance of the fluorescence of the newly synthesized protein in the endomembrane system (Figure 2B), and observations performed 48 h after transformation (Figure 2E) showed the Golgi stacks as the final localization of AB-GFP. Western blot analyses confirmed that the chimera predominantly accumulated intracellularly. Two pairs of bands migrating with an apparent molecular mass of approximately 34–36 and 30–32 kDa were, in fact, detected in the IN fraction (Figure 2C), with the lowest probably representing degradation forms of AB-GFP and the highest the entire chimera in agreement with its expected molecular mass (36 kDa). Both pairs of bands were faintly detected in the OUT fraction, likely due to default secretion, since their presence was drastically reduced in the presence of Sp2 (Figure 2D, Figures S5A–C). The persistence of the chimera in the Golgi stacks was confirmed in protoplasts co-expressing the AB-GFP fusion and ST52-mRFP, marker of the *trans* Golgi (Saint-Jore-Dupas et al., 2006). In the merged images, AB-GFP perfectly co-localized with ST52-mRFP (Figures 2E–H). Furthermore, the co-localization with the ER marker RFP-HDEL evidenced the transiting of the chimera in the ER, before reaching the Golgi (Figure S2).

Two potential *N*-glycosylation sites are predicted in B domain (NetNGlyc1.0 Server, www.cbs.dtu.dk/services/NetNGlyc/). To verify the presence of glycosylated forms of the chimera, AB-GFP-transformed protoplasts were treated with the *N*-glycosylation inhibitor tunicamycin (Elbein, 1987). After 24 h of incubation in the presence of the inhibitor, the upper band in each pair disappeared (Figure 2I) showing that AB-GFP and its degradation form were in part glycosylated in the ER.

To verify if the chimera AB-GFP was retained in the Golgi stacks through interactions with membranes, its solubility in the non-ionic detergent Triton X-114 was determined. After extraction and phase separation, we obtained an approximately equal distribution of the chimera in the detergent and aqueous phase, with the major percentage of the largest form in the detergent phase and of the smallest putatively degraded form in the aqueous phase (Figure 2J).

Confocal observations and biochemical data indicated that the construct AB-GFP entered in the secretory pathway, was glycosylated in the ER and mostly retained in the Golgi. These retained forms of the chimera appeared to be membrane-bound. The results also showed that the *N*-terminus prevents secretion of PGIP2 to the cell wall.

### The ABC truncated form of PGIP2 moves to the cell wall

**Figure 3:**
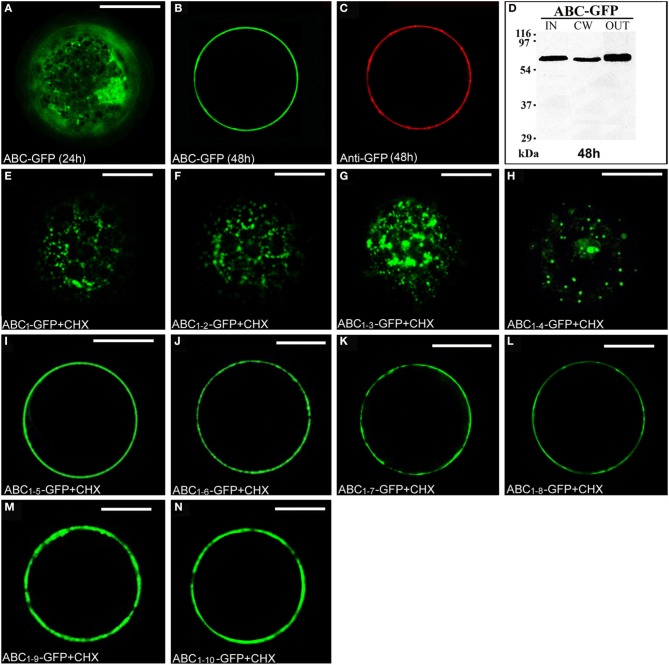
**The C domain allows ABC-GFP to reach the cell wall and the length of C sub-fragments determines a different cellular localization. (A,B)** ABC-GFP transiently expressed in tobacco leaf protoplasts progressively labeled ER, Golgi stacks **(A)** and cell wall **(B)**. **(C)** Immunolabeling of surface proteins was performed by incubating protoplasts with anti-GFP serum (3 h) and then with Alexa Fluor 546-conjugated anti-rabbit IgGs (1 h) at 4°C to minimize internalization of protein or antiserum (De Caroli et al., 2011a). The red fluorescence pattern was observed in 90 ± 4% among over 300 transformed protoplasts examined. **(D)** Western blot of protein fractions (intracellular, IN; cell wall, CW; incubation medium, OUT) obtained from ABC-GFP transformed protoplasts. **(E–H)** Chimeras containing from 1 to 4 LRRs of PGIP2 C domain localized in small punctate structures. **(I–N)** Constructs containing from 5 to 10 LRRs accumulated outside the cell. All tobacco leaf protoplast transformations were performed in the presence of cycloheximide (CHX) (30 μg · mL^−1^). All the fluorescent patterns were representative of more than 95% of the observed protoplast populations. Three independent transformation experiments were performed for each construct, and approximately 100 transformed protoplasts were counted each time. Bands were detected using anti-GFP serum. Scale bars = 20 μm.

The chimera ABC-GFP (Figure S1) labeled ER and Golgi stacks, as evidenced by co-localization with RFP-HDEL (Figures S3A–D) or ST52-mRFP (Figures S3E–H) and, during time, the cell wall, as verified by immunolocalization with an anti-GFP antibody and Alexa Fluor 546-conjugated anti-rabbit IgGs (Figures 3A–C). A rapid regeneration of a new cell wall starting already 6 h after protoplast preparation is well documented (Leucci et al., 2007; Yang et al., 2008; De Caroli et al., 2011a,b). The new synthesized cell wall was therefore the final compartment in which ABC-GFP is accumulated and recognized by anti-GFP antibody. Immunoblot analyses confirmed confocal observations showing the presence of the chimera in the IN, CW, and OUT fractions (Figure 3D). Both experimental approaches showed that ABC-GFP behaves as the full-length PGIP2 protein (De Caroli et al., 2011a), indicating that C domain allows the protein to be soluble in the lumen of the Golgi stacks and to move to the cell wall. Similar to PGIP2-GFP, ABC-GFP secretion was not affected by Sp2 (Figure S4).

To investigate the role of C domain in the targeting of PGIP2 to the cell wall, it was fragmented in 10 sub-domains progressively containing from 1 to 10 LRRs, which were fused to GFP. The chimeras were named progressively from ABC_1_-GFP to ABC_*1–10*_-GFP (Figure S1). To directly check the final localization of the chimeras, transiently transformed tobacco protoplasts were treated with the protein synthesis inhibitor cycloheximide. After 2 h of treatment, small punctate structures, likely Golgi stacks, were the steady-state location of chimeras containing from 1 to 4 LRRs (Figures 3E–H). Constructs containing from 5 to 10 LRRs accumulated on the surface of the cells, likely into the cell wall (Figures 3I–N).

**Figure 4:**
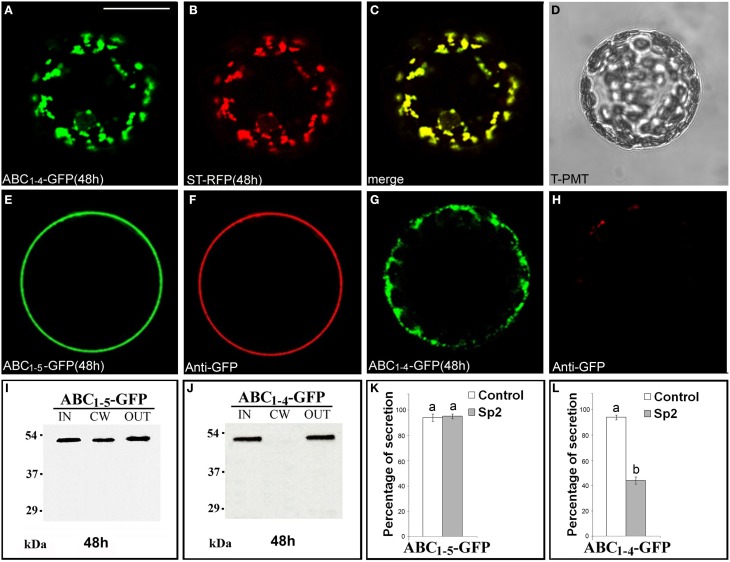
**ABC_***1–4***_-GFP is retained in Golgi stacks, while ABC_***1–5***_-GFP reaches the cell wall. (A–D)** 48 h after transformation, ABC_***1–4***_-GFP transiently expressed in tobacco leaf protoplasts labeled Golgi stacks **(A)**, as confirmed by co-localization with the Golgi marker ST52-mRFP **(B)**. **(C)** Merged image of **(A,B)**. **(D)** Transmitted light detector (T-PMT) image of **(A–C)**. The complete co-localization was observed in 89 ± 2% among over 300 transformed protoplasts examined. **(E–H)** Immunolabeling of surface protoplasts expressing ABC_***1–5***_-GFP **(E,F)** and ABC_***1–4***_-GFP **(G,H)** with anti-GFP serum and with Alexa Fluor 546-conjugated. The red fluorescence immunolabeling was observed in 89 ± 5% among over 300 transformed protoplasts examined. Three independent transformation experiments were performed for each construct, and approximately 100 transformed protoplasts were counted each time. **(I,J)** Western blot of protein fractions (intracellular, IN; cell wall, CW; incubation medium, OUT) obtained from tobacco protoplast transformed with ABC_***1–5***_-GFP **(I)** and ABC_***1–4***_-GFP **(J)**. **(K,L)** Quantification of the effect of Sp2 on the secretion of ABC_***1–5***_-GFP **(K)** and ABC_***1–4***_-GFP **(L)**. Statistical analysis is as in Figure 1. Bands were detected using anti-GFP serum. Scale bars = 20 μm.

Since ABC_*1–4*_-GFP was retained in punctate structures and ABC_*1–5*_-GFP instead reached the surface of transformed protoplasts, we focused on these two chimeras to define unambiguously their final localization. The punctate structures labeled by ABC_*1–4*_-GFP fully co-localized with ST52-mRFP marker confirming their nature as Golgi stacks (Figures 4A–D). The final localization of ABC_*1–5*_-GFP on the surface of transformed protoplasts was confirmed by immunolocalization with anti-GFP antibody and Alexa Fluor-546-conjugated anti-rabbit IgGs (Figures 4E,F). Only very weak non-specific signals were detected on the surface of protoplasts expressing ABC_*1–4*_-GFP (Figures 4G,H), clearly confirming the intracellular retention of this chimera.

Western blot analyses of proteins present in IN, CW, and OUT fractions confirmed the presence of ABC_*1–5*_-GFP (Figure 4I) and the absence of ABC_*1–4*_-GFP (Figure 4J) in the cell wall and the presence of both chimeras, which displayed the expected molecular size, in the incubation medium. Sp2 did not affect the secretion of ABC_*1-5*_-GFP in the cell wall and in the incubation medium (Figure 4K, Figures S5J–L), while drastically inhibited the amount of ABC_*1–4*_-GFP in the medium (Figure 4L, Figures S5D–F), indicating that the secretion of this chimera occurs via a default pathway.

### PGIP2 and its B domain-deleted form move through different mechanisms

**Figure 5:**
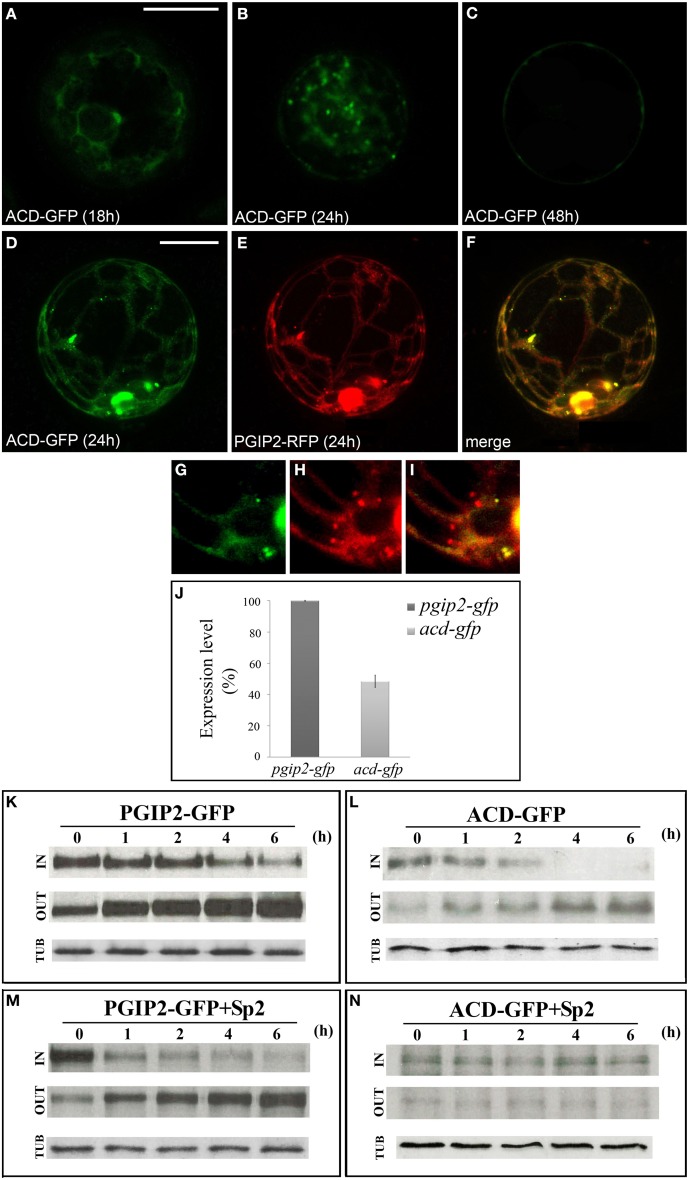
**PGIP2 depleted of B domain (ACD-GFP) was expressed at low level and secreted by default. (A)** ACD-GFP progressively labeled ER, **(B)** Golgi stacks, and **(C)** the cell wall. **(D–I)** Three-dimensional (3D) images of protoplasts co-expressing ACD-GFP **(D,G)**, PGIP2-RFP **(E,H)** and merged image of **D** and **E (F,I)**. Not all punctate structures are labeled by PGIP2 (red) and its B domain-depleted form (green) **(G-I)**. The fluorescent pattern was observed in 79 ± 8% among over 300 transformed protoplasts examined. Three independent transformation experiments were performed, and approximately 100 transformed protoplasts were counted each time. **(J)** Quantification of the amount of *pgip2-gfp* and *acd-gfp* expression level. A Real-time PCR was performed on protoplasts expressing PGIP2-GFP and ACD-GFP, the amount of *pgip2-gfp* and *acd-gfp* was quantified relative to the control actin RNA transcript. **(K,L)** Pulse-chase experiment of PGIP2-GFP and ACD-GFP secretion in transiently transformed protoplasts. Differently from PGIP2-GFP secretion **(M)**, secretion of ACD-GFP was affected by Sp2 **(N)**. Transformed protoplasts were pulse-labeled for 2 h and chased for the indicated periods of time. Tubulin (TUB) was used as an internal control.

Since the results obtained suggest a critical interplay between B and C domains in determining trafficking of PGIP2 to the wall, to further clarify the role of the B domain, we constructed a fluorescent PGIP2 chimera deleted of this sequence, hereon indicated as ACD-GFP (Figure S1). Similar to PGIP2-GFP, ACD-GFP moved through the endomembrane system labeling ER, Golgi stacks and cell wall (Figures 5A–C). However, the intensity of the ACD-GFP signal in the protoplasts and of the ACD-GFP bands on Western blot appeared much lower than that of PGIP2-GFP. This difference was likely due to differences in transcript levels, since levels of *acd-gfp* transcripts were about 50% that those of *pgip2-gfp* (Figure 5J), as evaluated in transformed protoplasts by quantitative RT-PCR.

To verify the overlapping of the secretion route of the two chimeras, we constructed a red fluorescent variant of PGIP2 (PGIP2-RFP) and transiently expressed both PGIP2-RFP and ACD-GFP in tobacco protoplasts (Figures 5D–F). Interestingly, some punctate structures resulted labeled only by PGIP2-RFP (Figures 5G–I), a difference that may be explained by the lower expression of ACD-GFP with respect to PGIP2-RFP, or, considering the previous indication that the B domain alone is retained in the Golgi, by a higher secretion rate of the B domain-deleted construct.

To overcome the problem of the different expression levels of the fluorescent ACD-GFP and PGIP2-GFP, the comparison between the secretion of these two proteins was performed by immunoprecipitation after pulse-chase labeling for 2 h with a [^35^S] protein labeling mixture, following the methodological approach of Frigerio et al. (1998). PGIP2-GFP synthesized within a 2 h pulse was progressively secreted into the medium during the following 6 h chase (Figure 5K), and its secretion pattern was not changed by the presence of Sp2 (Figure 5M). No detectable bands were observed in the CW protein fraction up to 6 h chase. Our results are in agreement with previous observations showing that PGIP2-GFP labels protoplast cell wall approximately 48 h after transformation and its secretion is unaffected by Sp2 (De Caroli et al., 2011a). In agreement with the lower expression level, the intensity of ACD-GFP bands was weak. However, the distribution pattern of the chimera in the IN and OUT fractions clearly showed that, unlike PGIP2-GFP, ACD-GFP was completely secreted within 4 h (Figure 5L) and clearly accumulated intracellularly in the presence of Sp2 (Figure 5N). These data indicate that the B domain-deleted PGIP2 form is secreted via a default pathway more rapidly than the full-length PGIP2 and suggest a critical role of the B domain in the Sp2-independent secretion of PGIP2.

### C domain length affects the solubility of the chimeras

**Figure 6:**
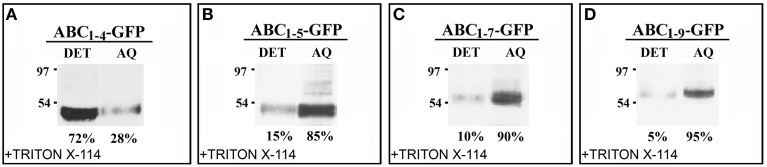
**The length of C domain modulates PGIP2 chimera membrane interaction. (A–D)** Detergent extraction in Triton X-114 of transformed protoplasts showing the progressive increase of soluble nature of constructs containing 1–5, 1–7, and 1–9 LRRs of C domains. Bands were detected using anti-GFP serum.

The effect of C domain length on the interaction of the chimeras with Golgi membrane was also investigated. For this purpose, protein extraction and phase separation in the presence of Triton X-114 was performed. Starting from the observations that AB-GFP was identified as a membrane-interacting protein (Figure 2J) and PGIP2-GFP was unable to bind to membranes (De Caroli et al., 2011a), we chose to analyze the chimeras ABC_*1–4*_-GFP and ABC_*1–5*_-GFP, representing respectively the longest construct retained in Golgi stacks and the shortest to be released (Figure 4), and the two chimeras carrying 7 and 9 LRRs (ABC_*1–7*_-GFP, ABC_*1–9*_-GFP), which both reached the cell wall (Figures 3K,M). The detergent and aqueous distribution of the chimeras clearly showed the membrane-interacting behavior of ABC_*1–4*_-GFP, it was in fact mainly recovered in the detergent phase (72%) (Figure 6A), and the soluble nature of the forms carrying 5, 7, and 9 LRRs found, on the contrary, in the aqueous phase (Figures 6B–D). Interestingly, the amount of proteins recovered in the aqueous phase increased with the length of C domain.

**Figure 7:**
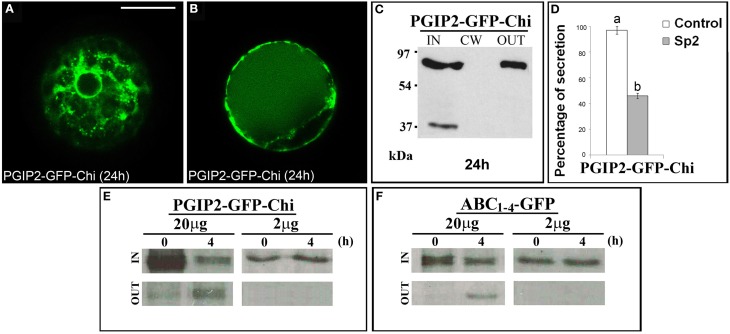
**PGIP2-GFP-Chi reaches the vacuole, a saturable mechanism is at the basis of PGIP2-GFP-Chi vacuolar sorting as well as of the Golgi retention mediated by B domain**. **(A)** PGIP2-GFP-Chi transiently expressed in tobacco leaf protoplasts progressively labeled ER, Golgi stacks and **(B)** the large central vacuole. The same fluorescent pattern was observed in 96 ± 4% among over 300 transformed protoplasts examined (100 for each of three independent transformations). **(C)** Western blot of protein fractions (intracellular, IN; cell wall, CW; incubation medium, OUT) obtained from tobacco protoplast transformed with PGIP2-GFP-Chi. **(D)** Quantification of the inhibitory effect of Sp2 on the secretion of PGIP2-GFP-Chi into the medium. Statistical analysis is as in Figure 1. **(E,F)** Intracellular and incubation medium protein fractions obtained from tobacco protoplasts transiently transformed with 20 or 2 μg of the plasmid encoding PGIP2-GFP-Chi **(E)** or ABC_*1–4*_-GFP **(F)**, pulse-labeled for 1 h with a protein labeling mixture [^35^S] and chased for the indicated periods of time. Scale bars = 20 μm.

The data obtained with the C domain suggest the presence in this sequence of regions/signals that allow the protein to leave the Golgi. To ascertain if these regions/signals can also act as cell wall sorting signals, we fused a vacuolar targeted GFP, GFP-Chi (Di Sansebastiano et al., 1998) to the full-length PGIP2 protein. The obtained fluorescent chimera PGIP2-GFP-Chi transiently expressed in tobacco protoplasts labeled the ER and Golgi stacks and the central vacuole (Figures 7A,B) without ever labeling the cell wall. Western blot clearly showed the presence of the chimera in the IN and OUT but, again, not in the CW fractions (Figure 7C). The capability of the Chi signal to divert the path of PGIP2 from the cell wall to the vacuole corroborates the idea that the C domain is a releasing rather than a cell wall sorting signal. The meaning of the presence of the chimera in the medium was analyzed as described below.

### Membrane interaction mediated by the B domain is saturable

It has been reported that, in transiently transformed protoplasts, the vacuolar sorting is a saturable mechanism and that saturation leads to default secretion of the exceeding protein to the incubation medium (Frigerio et al., 1998). In agreement with this notion, PGIP2-GFP-Chi was detected also out of the cells (Figure 7C); moreover, its secretion was inhibited by Sp2 (Figure 7D, Figures S5G–I) suggesting a default pathway. Similar to PGIP2-GFP-Chi, we observed that all the truncated forms retained in the Golgi were always in part detected in the medium and that, also in this case, secretion was affected by Sp2 (Figures 2C,D; Figures S5A–C, Figures 4J,L; Figures S5D–F). To search for a saturable retention mechanism similar to that operating in vacuolar sorting, we transformed protoplasts with either PGIP2-GFP-Chi or ABC_*1–4*_-GFP, using lower amounts of plasmid DNA (2 μg, instead of the standard amount of 20 μg, for approximately 10^6^ protoplasts). Transformed protoplasts were then pulse-labeled for 1 h with a [^35^S] protein labeling mixture and chased for 4 h. When expressed at low level, both PGIP2-GFP-Chi and ABC_*1–4*_-GFP were still present in the intracellular fraction, but none of them was detected in the incubation medium (Figures 7E,F). These results suggest that the default secretion of the Golgi-retained construct ABC_*1–4*_-GFP depends on the saturation of the B-domain interacting Golgi system. The saturation mechanism appears similar to that reported for vacuolar secretion, as observed in our experimental system with the construct PGIP2-GFP-Chi.

## Discussion

PGIP2 reaches the cell wall moving as soluble cargo along the secretory pathway through mechanisms distinguishable from a default sorting (De Caroli et al., 2011a). In this work we have provided evidence on specific roles of the main domains of PGIP2 previously described by Leckie et al. (1999) as: A, signal peptide; B, *N*-terminal; C, leucine-rich repeats.

### A domain is the signal peptide of PGIP2

The presence of a *N*-terminal specific signal is a feature of many secreted proteins regardless of whether or not they transit through the conventional secretion pathway. The first 20 aa at the *N*-terminus of PGIP2 (A domain, Figure S1) were postulated to be a putative signal peptide (Leckie et al., 1999). Our analyses on the GFP-tagged A domain showed that it allows translocation into ER and secretion into the medium with a pattern similar to the default secretion of the synthetic protein secGFP (Batoko et al., 2000; Leucci et al., 2007). Both chimeras are sensitive to Sp2, the dominant negative truncated form of SYP121, known to interfere with the default *post*-Golgi transport mediated by this syntaxin (Geelen et al., 2002; Leucci et al., 2007). Therefore, the *N*-terminal A domain of PGIP2, like the sec signal of secGFP, represents the classical signal peptide (SP) necessary for protein translocation into ER and for protein secretion through the ER/Golgi secretory pathway. No other signals are present in this domain since the chimera was secreted into the medium by default.

### Golgi holding signals are present in B domain

The construct AB-GFP, consisting of the first 68 aminoacids of PGIP2, including the signal peptide (A domain) and the presumed *N*-terminus of the mature protein (B domain), labeled ER and Golgi. Golgi stacks are the stable and final localization of the chimera as evidenced by co-localization with the Golgi marker ST52-mRFP and the disappearance of fluorescence from ER in the presence of cycloheximide. The construct AB-GFP was detected in the intracellular fraction proteins in a glycosylated and non-glycosylated form, confirming its insertion in the secretory pathway and its transit in the ER.

PGIP2 moves through the secretion pathway as soluble cargo without any interaction with membranes (De Caroli et al., 2011a). Differently from the full-length protein, after extraction and phase separation in the presence of Triton X-114, the chimera AB-GFP was detected in the detergent phase suggesting its interaction with the endomembrane system. No putative signals indicative of membrane binding (GPI-anchors or TM sequences) are predicted in B domain (predGPI software, TMHMM server), which is also hydrophilic. Therefore, the interaction of B domain with Golgi membranes could be: (i) an intermediate step in PGIP2 secretion to the cell wall or (ii) an aspecific process due to the presence of sites exposed in the fragment B but not in the full-length PGIP2 protein; a series of experimental evidence are in favor of the first hypothesis. When the full-length C domain, consisting of 10.5 LRRs was added to the AB fragment, the chimera ABC-GFP was no longer retained in the Golgi stacks and reached the cell wall. Significantly, this release occurred starting from the addition of the fifth LRR, while the constructs containing the first four LRRs remained stably held in the Golgi. A switch from membrane-interacting constructs to soluble forms occurred only after the addition of the fifth LRR, which does not establish interactions with the B domain (Di Matteo et al., 2003) (Figures 3, 6). It is therefore difficult to explain these results with an accidental generation of both retention and export signals. On the contrary, it is reasonable to hypothesize the presence of specific informations in C sub-fragments that overcomes the effect of the B domain, allowing the protein to leave Golgi and continue its journey toward the cell wall. Therefore, the dissection of PGIP2 in its domains and the fragmentation of the C domain in sub-fragments likely allows to unmask the potential of the B domain to interact with Golgi membranes that is not detectable during the trafficking of the full-length protein.

Multiple signals seem to be involved in the segregation of Golgi resident proteins from secretory traffic to different compartments of Golgi stacks. TM domains are considered to provide a dominant localization signal, but contributions from cytoplasmic tails and lumenal domains have also been reported (Munro, 1995; Brandizzi et al., 2002; Hawes, 2005; Schoberer and Strasser, 2011; Chevalier et al., 2014). The lumenal domain of some glycosyltransferases seems to have a targeting role by promoting interaction with existing membrane complexes. A soluble form of *N*-acetylglucosaminyl-transferase I (Glc*N*ac-T1), carrying a lumenal domain but lacking the TM domain, accumulates in the Golgi prior to secretion. The retardation of the soluble form within Golgi is related to the inclusion into membrane-linked high molecular weight complexes, whose nature remains obscure (Opat et al., 2000). Therefore, if the lumenal domain may be important for the targeting of proteins that also possess TM domains, it is even more plausible that soluble proteins that must be addressed to particular regions of Golgi stacks possess specific regions that act as signals. These may allow the proteins to be sequentially modified or selectively segregated for trafficking routes different from the default pathway. With respect to this, interesting results on cell wall protein trafficking have been reported. Dal Degan and co-workers (2001) reported that an endopolygalacturonase 1 is temporarily stored inside the cell until triggered to secretion by a sorting signal within the cleavable *N*-terminal domain. Furthermore, evidence has been provided for the involvement of the *N*-terminal domain, the PRO region, in the temporary retention of pectin methylesterase (PME) isoforms within the Golgi stacks, through the binding with a resident interactor (likely an inactive protease or unknown auxiliary factors). Proteolytic release of the PRO region, occurring in the Golgi, is a pre-requisite for apoplastic targeting of PME (Dorokhov et al., 2006; Wolf et al., 2009a,b). Although studies on cell wall protein and polysaccharide trafficking are still very few, it is significant that independent researchers reported specific controls on cell wall protein sorting, all related to a transient holding in the Golgi.

To corroborate the hypothesis that the B domain of PGIP2 plays a role in the trafficking of the protein, likely determining a transient interaction with Golgi membranes, a PGIP2 fluorescent chimera deleted of this domain (ACD-GFP) was constructed and its trafficking was compared to that of full-length PGIP2 (ABCD-GFP). Interestingly, GFP signal intensity was clearly lower in protoplasts transformed with B-depleted construct than PGIP2 transformed cells. Analyses of PGIP2-GFP and ACD-GFP gene expression clearly showed that levels of the ACD-GFP transcripts were lower by about 50%, suggesting a role of the region encoding the B domain in transcript stability or a role of the B domain in PGIP2 expression. Pulse-chase analyses clearly showed different secretion kinetics of the two chimeras. While ACD-GFP was completely secreted within a 4 h chase, PGIP2-GFP was continuously secreted over a 6 h chase. Furthermore, unlike PGIP2, ACD-GFP secretion was drastically affected by Sp2, indicating the involvement of a default pathway (Geelen et al., 2002; Rehman et al., 2008). These results confirm that PGIP2 trafficking toward the cell wall does not occur by default (De Caroli et al., 2011a). They also support the hypothesis of the presence in the B domain of a holding signal that may slow down PGIP2 delivery to the cell wall. Finally, they provide evidence that these signals regulate PGIP2 sorting since, when they are lacking, protein secretion is converted from a controlled route to a default pathway. Unregulated passive inclusion of B-deleted PGIP2 into vesicles may occur more rapidly in the absence of interaction with auxiliary factors present on the Golgi membranes.

### The C domain contains Golgi-releasing information

In the ABC-GFP construct, as well as in the full-length PGIP2, the presence of the C domain allows the protein to leave Golgi and to move to the cell wall (Figure 3). The C domain seems to act as a releasing signal rather than as a classical sorting target as evidenced with the construct PGIP2-GFP-Chi, where the vacuolar sorting signal Chi was added to PGIP2. The fluorescent vacuolar reporter GFP-Chi, carrying a *C*-terminal sorting determinant of tobacco chitinase (Di Sansebastiano et al., 1998), is widely used to study vacuolar trafficking as well as vacuole biogenesis (Flückiger et al., 2003; Stigliano et al., 2013). A direct ER-to-vacuole transport has been recently described that does not involve the COPII transport machinery and is independent from Golgi and *post*-Golgi trafficking (Viotti et al., 2013; Viotti, 2014), and GFP-Chi has been reported as a specific marker of a Golgi-independent traffic to the central vacuole (Faraco et al., 2013; Stigliano et al., 2013, 2014). Our construct PGIP2-GFP-Chi labeled Golgi stacks indicating that, in the full-length PGIP2, signals for a conventional ER to Golgi transport are dominant on Chi-dependent mechanisms. However, unlike PGIP2-GFP, PGIP2-GFP-Chi accumulates into the central vacuole indicating that Chi is a more strong recognizable signal than the information in the C domain, later acting as a dominant vacuolar sorting signal. Likely, Chi signal recognition in the Golgi stacks depends on versatility of vacuolar sorting recognition sites (Suen et al., 2010; De Marcos Lousa et al., 2012).

The distribution ratio between the detergent (membrane interacting) and aqueous (soluble) phases of the constructs containing 1–4, 1–5, 1–7, and 1–9 LRRs of C domain also provides interesting information. The amount of protein recovered in the aqueous phase increased with the length of C domain (Figures 6A–D). By analyzing the trafficking of each PGIP2 domain in conditions of high expression, which may reveal events not appreciable in normal expression conditions, it was possible to observe: (i) a stable membrane interaction of the chimera containing only the B domain (Figure 2J); (ii) a complete solubility of the full-length PGIP2 (De Caroli et al., 2011a); (iii) intermediate features for the chimeras containing increasing length C sub-fragments (Figures 6A–D). All together the results suggest that trafficking of PGIP2 to the cell wall likely occurs through complex mechanisms related to specific interactions with Golgi membranes involving hold/release steps specifically mediated by B and C domains.

### Golgi membrane retention of B domain is a saturable mechanism

We have discussed above that the sorting to the cell wall likely occurs through complex interactions with Golgi membranes involving a retention step mediated by the B domain and, likely, unknown auxiliary factors. The evidence that the system mediating retention is saturable supports the existence of these factors. The constructs AB-GFP and ABC_*1–4*_-GFP, retained in the Golgi stacks, were always also detected in the incubation medium (OUT) (Figures 2C, 4J); significantly, the percentage of the chimeras detected outside the cell drastically decreased in the presence of Sp2 (Figures 2D, 4L), clearly indicating a secretion via default. A saturable membrane interaction mechanism may explain this default secretion. When the capacity of unknown auxiliary factors to interact with B domain is saturated (as in conditions of high expression) the excess of chimera escapes controls and is secreted by default, passively enclosed into vesicles. On the contrary, secretion of the full-length PGIP2 to the apoplast, as well as of the constructs containing the entire or a large portion (≥5 LRRs) of the C domain, is never affected by Sp2 (Figure S4; Figures 4K, 5M), excluding the involvement of the default pathway. As discussed above, when both B and C domains are present, membrane interactions involving transient hold/release steps likely occur, the transient nature of this interactions not allowing the saturation of the mechanism.

The existence of a saturable targeting mechanism involved in the secretion of the vacuolar phaseolin has been evidenced by comparing high and low amounts of plasmid DNA in transiently transformed protoplasts. In a condition of phaseolin over-expression, the vacuolar sorting capacity of protoplasts was saturated and the excess of protein was secreted into the incubation medium via a default pathway. At lower levels of protein expression, however, no default secretion into the medium occurred (Frigerio et al., 1998). Adopting the same experimental strategy, we detected no secretion into the medium for the truncated PGIP2-GFP construct that, when expressed at high level, showed instead both retention in the Golgi and default secretion (Figure 7F). Further, as for phaseolin, PGIP2-GFP-Chi showed Sp2-sensitive secretion into the medium, and, at low amount of plasmid DNA (2 μg), was completely targeted to the vacuole (Figure 7E).

It is important to underline that the saturation of the vacuolar sorting machinery is easily evidenced because the exceeding vacuolar proteins are diverted in a different compartment, i.e., the apoplast, by default. Saturation of regulated protein secretion to the wall is more difficult to study because it overlaps with the default pathway. The inhibition of PGIP2-GFP-Chi secreted into the medium by Sp2 corroborates the validity of our system.

Altogether the evidence reported here, in addition to those previously reported in other studies (Dal Degan et al., 2001; Dorokhov et al., 2006; Leucci et al., 2007; Wolf et al., 2009a,b; De Caroli et al., 2011a,b), reveals a great complexity in the delivery of cell wall materials and the regulation of their Golgi trafficking. The heterogeneity of these materials, polysaccharides and both structural and enzymatic proteins, does not help to solve the problem. A classical sorting signal, as we expected on the basis of our knowledge on vacuolar sorting sequences, cannot be present in cell wall polysaccharides and, as reported in this work, seems to lack in cell wall proteins. The absence of such a specific cell wall sequence targeting and the difficulty to discriminate among two paths following the same direction (as above discussed) are likely the reasons that led to assumption, until recently, that the trafficking to the apoplast is a default pathway. Lately, the idea of more complex and highly regulated pathways is progressively growing up (Worden et al., 2012; Kim and Brandizzi, 2014). The evidence reported in this paper in favor of a controlled export of a cell wall protein that moves as soluble cargo adds new elements in this direction.

### Conflict of interest statement

The authors declare that the research was conducted in the absence of any commercial or financial relationships that could be construed as a potential conflict of interest.

## Supplementary material

Figure S1 **Schematic representation of the constructs analyzed in this study**. Asterisks indicate the putative glycosylation sites. Numbers mark location of the amino acids.

Figure S2 **Co-localization of AB-GFP with the ER marker RFP-HDEL**.

Figure S3 **ABC-GFP progressively labeled ER (A–D) and the Golgi stacks (E–H) before reaching the cell wall, as indicated by co-localization with the ER marker RFP-HDEL and the Golgi marker ST52-mRFP**.

Figure S4 **Quantification of the effect of Sp2 on the secretion of ABC-GFP**.

Figure S5 **The saturation of the retention mechanism mediated by B domain leads to default secretion affected by Sp2**. The default secretion of the Golgi-retained constructs AB-GFP and ABC_1–4_-GFP was inhibited in the presence of Sp2 **(A–F)** as well as the default secretion to the medium of the PGIP2-GFP-Chi **(G–I)**. No Sp2 effect was detected on ABC_1–5_-GFP secretion **(J–L)**.

Table S1 **Oligonucleotides used for cloning and qRT-PCR**.
