# Supplementary figures and images for: Molecular dissection of Phaseolus vulgaris polygalacturonase-inhibiting protein 2 reveals the presence of hold/release domains affecting protein trafficking toward the cell wall
Source: Front Plant Sci. 2015 Aug 26;6:660. doi: 10.3389/fpls.2015.00660 (PMC4550104; doi:10.3389/fpls.2015.00660)

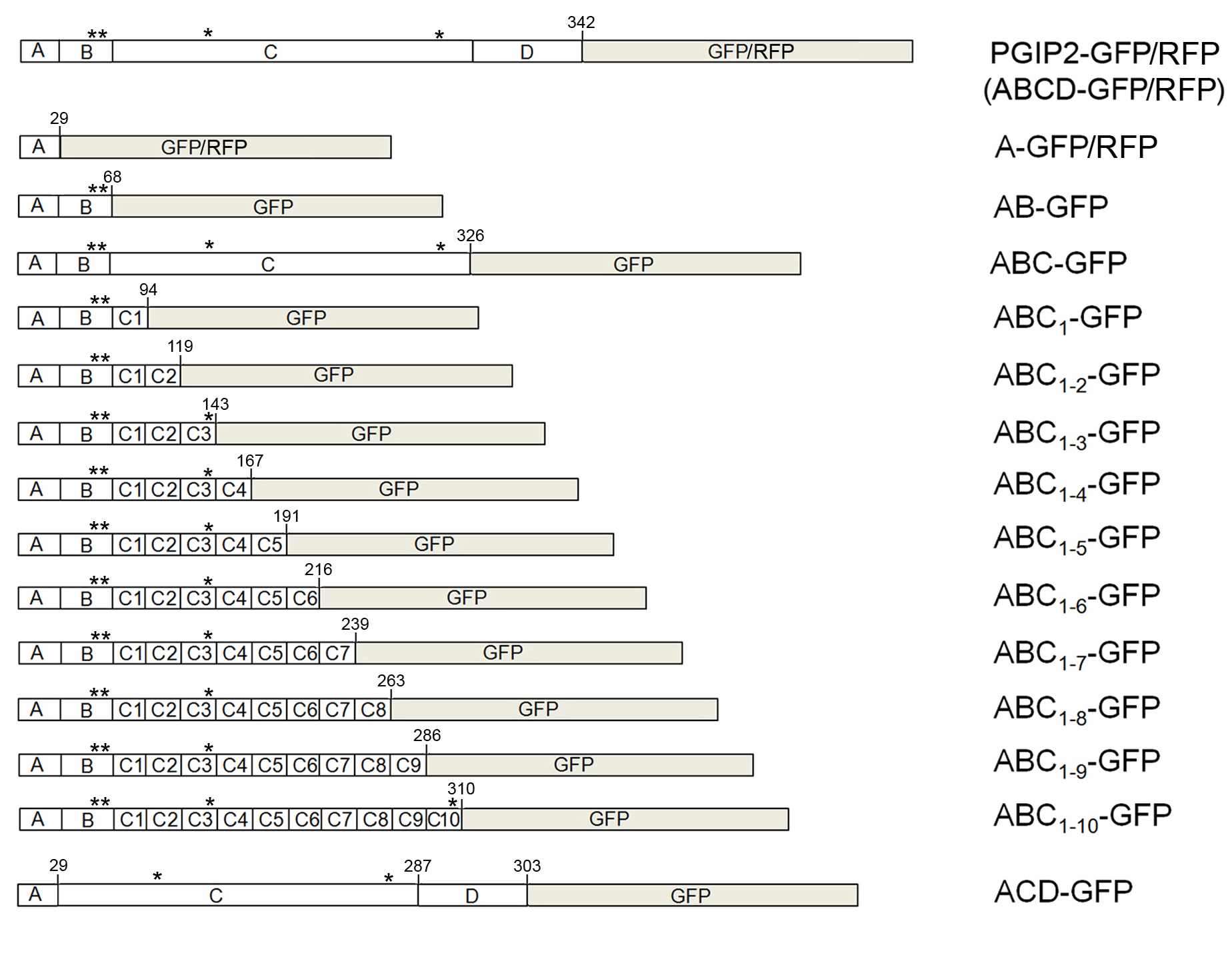

Supplement: Figure S1 — Schematic representation of the constructs analyzed in this study. Asterisks indicate the putative glycosylation sites. Numbers mark location of the amino acids. [file Image1.JPEG]

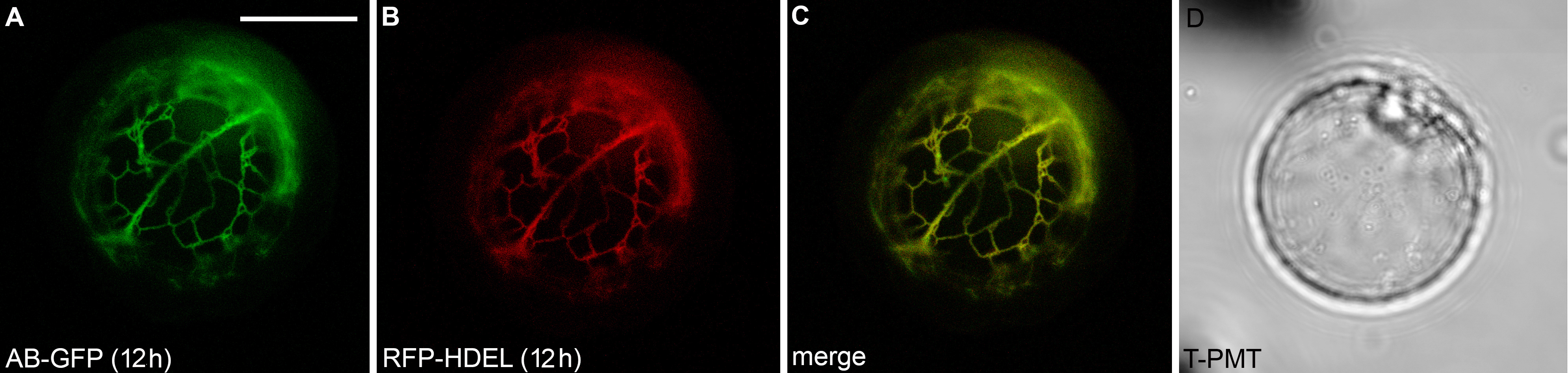

Supplement: Figure S2 — Co-localization of AB-GFP with the ER marker RFP-HDEL. [file Image2.JPEG]

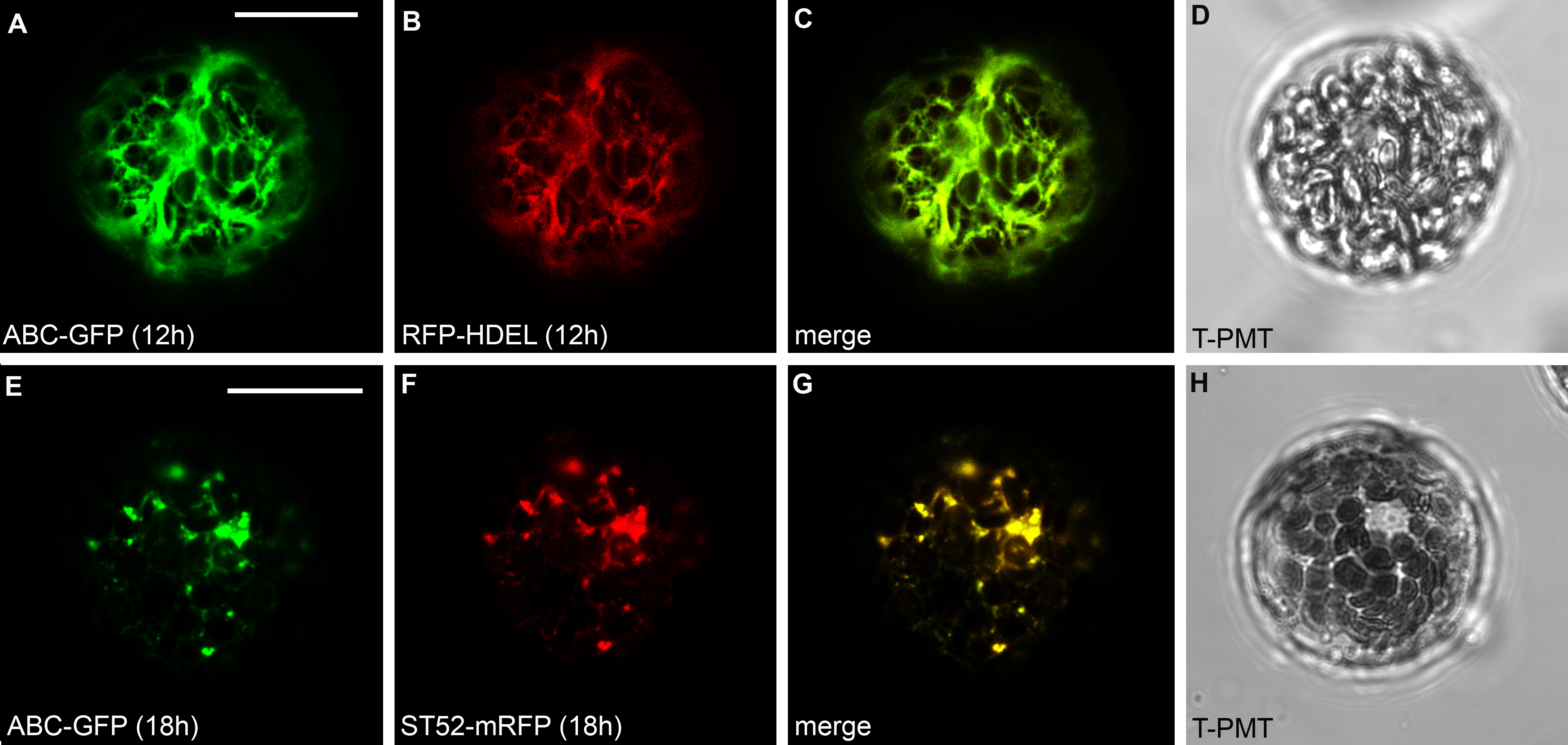

Supplement: Figure S3 — ABC-GFP progressively labeled ER (A–D) and the Golgi stacks (E–H) before reaching the cell wall, as indicated by co-localization with the ER marker RFP-HDEL and the Golgi marker ST52-mRFP. [file Image3.JPEG]

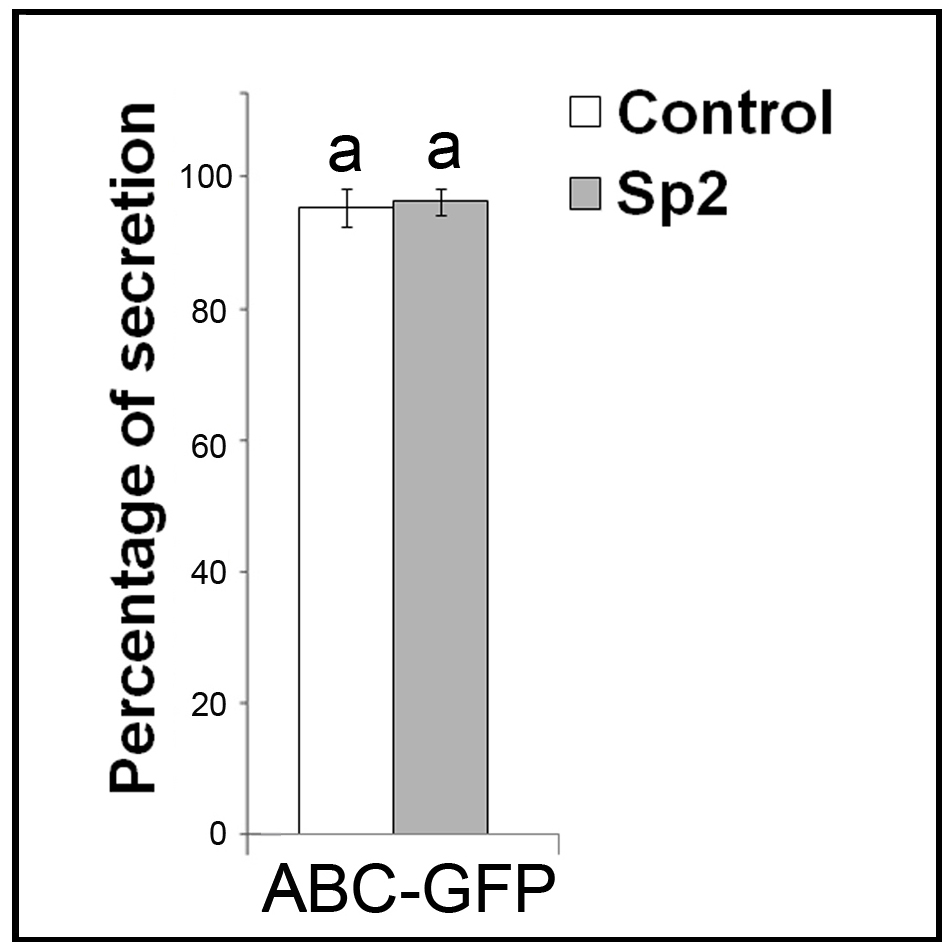

Supplement: Figure S4 — Quantification of the effect of Sp2 on the secretion of ABC-GFP. [file Image4.JPEG]

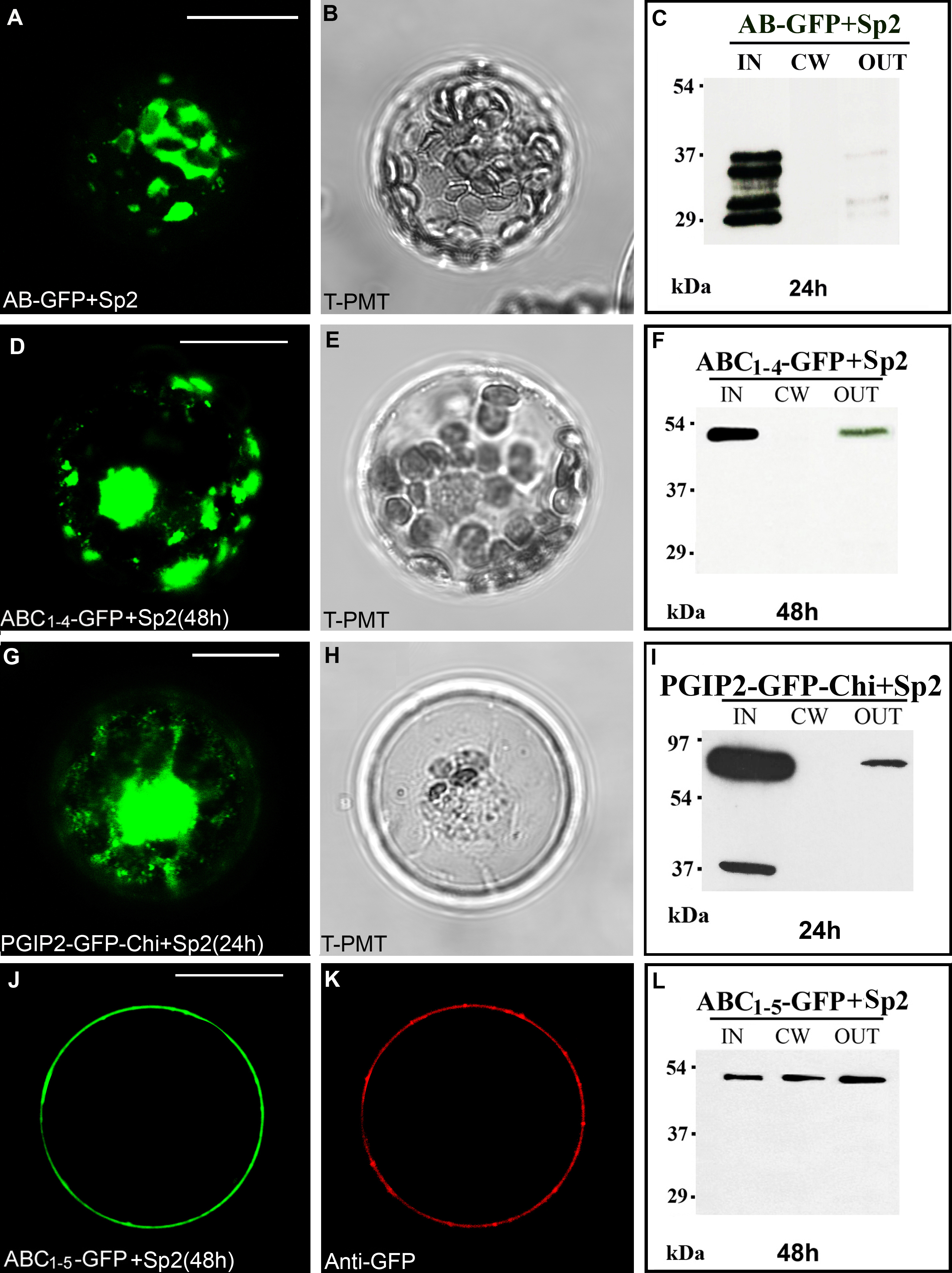

Supplement: Figure S5 — The saturation of the retention mechanism mediated by B domain leads to default secretion affected by Sp2. The default secretion of the Golgi-retained constructs AB-GFP and ABC1–4-GFP was inhibited in the presence of Sp2 (A–F) as well as the default secretion to the medium of the PGIP2-GFP-Chi (G–I). No Sp2 effect was detected on ABC1–5-GFP secretion (J–L). [file Image5.JPEG]
