# Supplementary material for: Molecular dissection of Phaseolus vulgaris polygalacturonase-inhibiting protein 2 reveals the presence of hold/release domains affecting protein trafficking toward the cell wall
Source: Front Plant Sci. 2015 Aug 26;6:660. doi: 10.3389/fpls.2015.00660 (PMC4550104; doi:10.3389/fpls.2015.00660)
Supplement: Table S1 — Oligonucleotides used for cloning and qRT-PCR. [file Table1.PDF]

Table S1

	name	sequences (5'-3')
P1	A (for)	gtatctttgagcactgcacactcagctagctgcaacccacaagacaagcaa
P2	A (rev)	ttgcttgtcttgtgggttgcagctagctgagtgtgcagtgctcaaagatac
P3	B (for)	gcaacagaacctggctagctagcttatgcgacaccgacaccc
P4	B (rev)	gggtgtcggtgtcgcataagctagctagccaggttctgttgc
P5	Pgipa	cagtcgctagtggatccatgact
P6	C (rev)	gttgttgctagcagcagaaacg
P7	C1 (rev)	gatgctagcgggttttgggag
P8	C2 (rev)	ggggatgctagcgacgagg
P9	C3 (rev)	cgggtatgctagcggagac
P10	C4 (rev)	gggaggtaggctagcggagag
P11	C5 (rev)	ggggatgctagcggagattcgg
P12	C6 (rev)	cggaatgctagcggtgaggcgg
P13	C7 (rev)	ccgacgcgctagcctccagc
P14	C8 (rev)	ccccaagctagcggcaagag
P15	C9 (rev)	gcggtaggctagcatagatacgg
P16	C10 (rev)	ccttgaggaatgctagcgcacag
P17	C7for	gacgcgtcggtgttgttcgg
P18	004	cagcgagttgcacgccgccg
P19	Nt-actfor1	tacaacgagcttcgtgttgc
P20	Nt-actrev1	ctctcggcaccaatggtaat
